# Expression of MicroRNAs in Adults with Celiac Disease: A Narrative Review

**DOI:** 10.3390/ijms25179412

**Published:** 2024-08-30

**Authors:** Francielen Furieri Rigo, Ellen Cristina Souza de Oliveira, Ana Elisa Valencise Quaglio, Bruna Damásio Moutinho, Luiz Claudio Di Stasi, Ligia Yukie Sassaki

**Affiliations:** 1 Department of Internal Medicine, Medical School, São Paulo State University (UNESP), Botucatu 18618-687, SP, Brazil; ellen.oliveira@unesp.br (E.C.S.d.O.); ligia.sassaki@unesp.br (L.Y.S.); 2 Verum Ingredients, Botucatu Technology Park, Botucatu 18605-525, SP, Brazil; ana.quaglio@verumingredients.com.br; 3 Department of Gastroenterology, Division of Clinical Gastroenterology and Hepatology, University of São Paulo School of Medicine (USP), São Paulo 01246-903, SP, Brazil; brunadmoutinho@gmail.com; 4 Laboratory of Phytomedicines, Pharmacology and Biotechnology (PhytoPharmaTec), Department of Biophysics and Pharmacology, Institute of Biosciences, São Paulo State University (UNESP), Botucatu 18618-689, SP, Brazil; luiz.stasi@unesp.br

**Keywords:** celiac disease, microRNAs, gluten-free diet, immune response, biomarkers

## Abstract

Celiac disease (CD) is an immune-mediated enteropathy triggered by the ingestion of proline- and glutamine-rich proteins, widely termed “gluten”, in genetically susceptible individuals. CD induces an altered immune response that leads to chronic inflammation and duodenal mucosal damage. Currently, there are no specific tests for the accurate diagnosis of CD, and no drugs are available to treat this condition. The only available treatment strategy is lifelong adherence to a gluten-free diet. However, some studies have investigated the involvement of microRNAs (miRNAs) in CD pathogenesis. miRNAs are small noncoding ribonucleic acid molecules that regulate gene expression. Despite the growing number of studies on the role of miRNAs in autoimmune disorders, data on miRNAs and CD are scarce. Therefore, this study aimed to perform a literature review to summarize CD, miRNAs, and the potential interactions between miRNAs and CD in adults. This review shows that miRNA expression can suppress or stimulate pathways related to CD pathogenesis by regulating cell proliferation and differentiation, regulatory T-cell development, innate immune response, activation of the inflammatory cascade, focal adhesion, T-cell commitment, tissue transglutaminase synthesis, and cell cycle. Thus, identifying miRNAs and their related effects on CD could open new possibilities for diagnosis, prognosis, and follow-up of biomarkers.

## 1. Introduction

Celiac disease (CD) is a common immune-mediated enteropathy triggered by the interaction between environmental and genetic factors. In genetically susceptible individuals carrying the human leukocyte antigen (HLA)-DQ2 or HLA-DQ8 haplotypes, ingestion of gluten present in wheat and similar proteins present in barley and rye induces an altered immune response, which leads to chronic inflammation because of the infiltration of a consistent number of lymphocytes and plasma cells in the lamina propria, followed by villous atrophy and crypt hyperplasia, resulting in duodenal mucosal damage [1,2].

Clinically, the disease may present with a wide spectrum of symptoms, such as diarrhea, abdominal pain, involuntary weight loss, bloating, fatigue, anemia, high transaminase levels, vitamin deficiencies, and infertility, resulting in a multisystem disorder rather than isolated intestinal disease [3,4]. CD diagnosis is based on the combined analyses of risk factors, clinical manifestations, serum antibodies, endoscopic findings, and histopathological duodenal lesions [5]. Although several drugs have been studied, the only available treatment strategy for CD is lifelong adherence to a gluten-free diet (GFD), which usually reverses symptoms and serological and intestinal alterations. However, GFDs are difficult to follow and may induce adverse metabolic effects, resulting in a reduction in the patient’s quality of life owing to this restrictive diet [6].

To date, the causes of CD have not been established, and there are no specific tests for their proper diagnosis and assessment. Some studies have investigated the involvement of microRNAs (miRNAs) in CD pathogenesis, suggesting that miRNAs may play important roles as CD biomarkers [7,8,9,10,11]. miRNAs are small, single-stranded, noncoding RNAs of approximately 18–25 nucleotides that can inhibit the translation of multiple messenger RNAs (mRNAs), thereby silencing their target genes. Despite the growing number of studies on the role of miRNAs in autoimmune disorders, data on miRNAs associated with CD are scarce.

Recently, circulating and intracellular miRNAs have been proposed as biomarkers for the diagnosis of CD and have potential applications in gene-based therapies [2,12]; however, their functions are not yet fully understood. Therefore, in this study, we aimed to provide the basic concepts of CD and miRNAs and review the current data on the relationship between CD and circulating and tissue miRNAs in adult patients.

## 2. Celiac Disease

CD is the most prevalent enteropathy worldwide [13]. In genetically susceptible individuals, ingestion of wheat gluten and similar proteins in rye and barley, commonly known as “gluten”, induces an immune-mediated response that results in inflammation and damages the small intestinal mucosa [1,2].

Historically, the disease has been considered to affect the pediatric population almost exclusively, with the “classic” presentation of diarrhea, malabsorption syndrome, poor growth, weight loss, and nutritional deficiencies. The diagnosis was based on a duodenal biopsy showing intense mucosal damage. Since the discovery of serological biomarkers, the active search for affected individuals with few gastrointestinal symptoms and “atypical” extraintestinal presentations and associated disorders has increased the number of adults being diagnosed [14].

Currently, CD is recognized worldwide, with histological and serological prevalence rates of approximately 1.4% and 0.7%, respectively [15]. CD can occur in most populations, regardless of age and sex, but its diagnosis is approximately twice more common in children than in adults, and it is 1.5 times more common in females than in males [15,16,17,18]. Most patients are diagnosed in developed countries, and a large number of patients with CD remain undiagnosed, particularly in developing countries [18,19].

Nearly all patients with CD carry a genetic risk; HLA-DQ2 or HLA-DQ8 haplotypes occur in at least 90–95% and 5–10% of patients with CD, respectively, although >40 non-HLA CD loci have been identified in genome-wide association studies [20,21]. The association between genetic factors was first described in a large observational study involving monozygotic twins that showed 70–75% concordance [22]. HLA-DQ2/DQ8 molecules are necessary, but not sufficient, for disease development, as HLA-DQ2/DQ8 haplotypes, for example, are carried by approximately 30–40% of healthy individuals, and only approximately 1% of the population develops CD [13,23,24].

**Figure 1:**
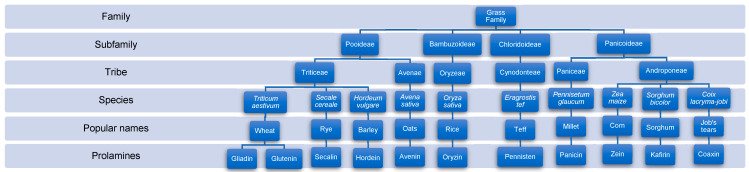
Phylogenetic relationships of some cereal species, names of family, subfamily, tribe, species, popular names, and their prolamines [25,26,27,28,29]. Flowchart generated using Microsoft Office 365 version 2408.

“Gluten” is a term commonly used to refer to specific prolamines, disease-activating proteins that trigger inflammation in CD, present in wheat (*Triticum* spp.), barley (*Horderum vulgare*), and rye (*Secale cereale*); however, gluten is the storage protein fraction present only in wheat grains [25,26]. Two primary prolamines are present in wheat gluten—glutenins and gliadins—which are both disease-activating peptides [25,26]. Wheat, barley, and rye belong to the same botanical tribe (Triticeae); therefore, they contain closely related proteins that trigger inflammation, including secalins in rye and hordeins in barley. Oats (*Avena sativa*), whose prolamines are avenins, are distantly related to wheat, barley, and rye and belong to another botanical tribe, Aveneae [26]. Overall, oat gluten is inoffensive to individuals with CD, but cross-contamination with other cereals during its processing and storage is commonly observed; therefore, it is recommended to avoid it [4]. The grass family (Poaceae) also includes rice, maize, sorghum, millet, Job’s tears, and teff, but they are phylogenetically distant and share only the family group; therefore, analogous proteins do not activate CD [25,26] (Figure 1).

Gliadins, glutenins, hordenins, and secalins have high proline and glutamine content, whereas avenins have low levels [25,27]. The high proline content makes these proteins resistant to complete proteolytic digestion, resulting in the accumulation of relatively large gluten peptide fragments in the small intestine [25,27,28,29]. In active disease, gluten degradation is more difficult than usual because of damage to the small intestinal mucosa [25].

Gluten peptides cross the epithelium into the lamina propria through two routes [13]. One of these mechanisms involves increased gut permeability by disrupting the tight junctions of enterocytes. Gluten peptides bind to upregulated chemokine receptors present at the apex of enterocytes in patients with CD, namely CXCR3, releasing zonulin, a protein that regulates intestinal permeability [13,30,31]. Zonulin is produced in the intestine and helps to control the opening of tight junctions between intestinal cells, allowing the passage of nutrients and other substances into the body. Thus, higher levels of CXCR3 in CD lead to increased levels of zonulin, which facilitates the paracellular translocation of gluten peptides to the lamina propria [13,30].

**Figure 2:**
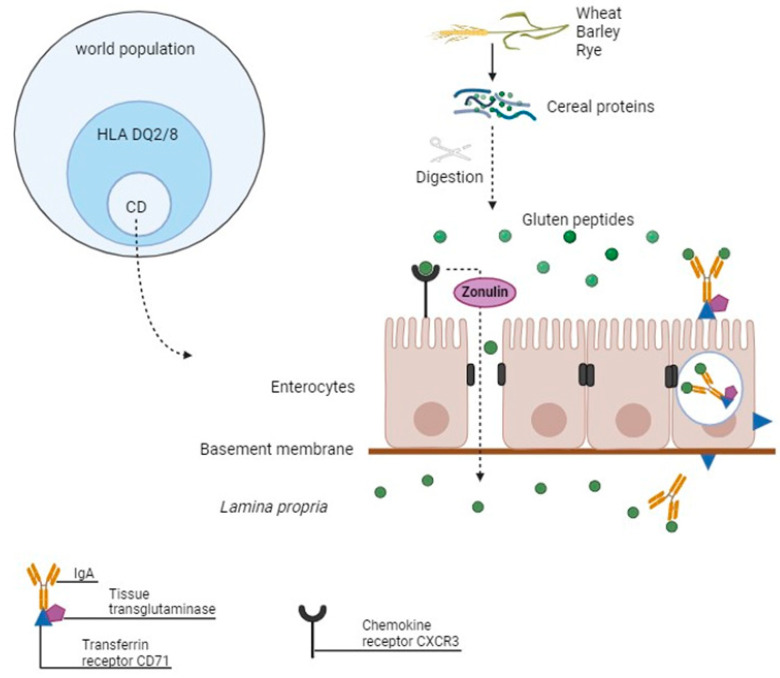
Simplified schematic representation of gluten peptide transport through the intestinal epithelium in patients with celiac disease. Up to 40% of healthy individuals carry human leukocyte antigen DQ2 or DQ8 haplotypes, but only 1% of the world population develops celiac disease (left diagram). During the digestion of wheat, barley, and rye, specific proteins called gluten release gluten peptides, which cross the basement membrane to the lamina propria in two ways: by the paracellular route and by retrotranscytosis. In the first mechanism, gluten peptides bind to the chemokine receptor CXCR3, which induces zonulin release with subsequent disassembly of tight junctions. In the second method, the peptides form a trimeric complex with secretory immunoglobulin A, transferrin receptor CD71, and tissue transglutaminase, which transfers the antigen from the apical to the basal side of the epithelium.

The second method involves retrotranscytosis of a complex formed by three components, namely tissue transglutaminase (tTG), secretory immunoglobulin A (IgA), and transferrin receptor CD-71, which are overexpressed in CD enterocytes [13,31]. In healthy individuals, “gluten” peptides are taken up nonspecifically by enterocytes and degraded by lysosomal acid proteases during transcytosis. Very few peptides are delivered to the intestinal lamina propria. In patients with active CD, abnormal expression of CD71 at the apical pole of enterocytes allows receptor-mediated uptake of secretory IgA-“gluten” peptide complexes and their protected transport toward the lamina propria, and thus toward the local immune system [32](Figure 2).

**Figure 3:**
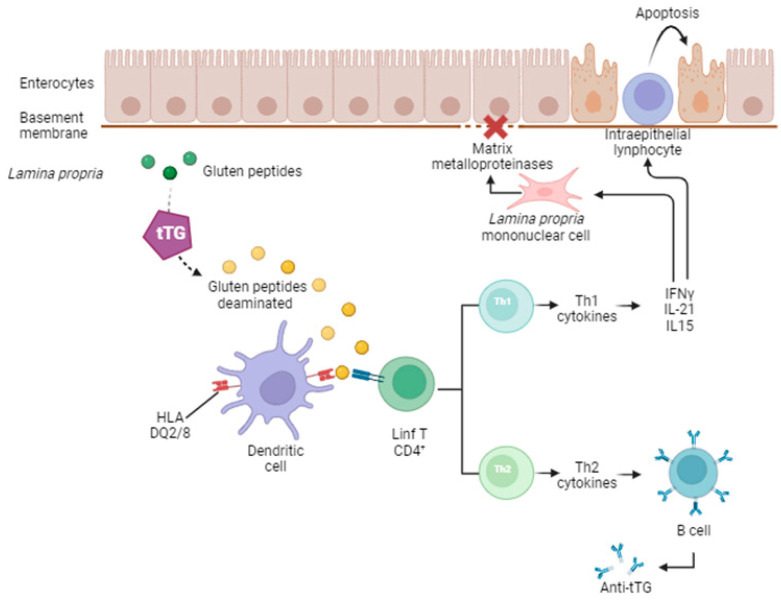
Simplified schematic representation of celiac disease pathogenesis. Once gluten peptides reach the lamina propria, they undergo deamination (dotted arrow) by tissue transglutaminase (tTG). Deaminated gluten peptides are rich in negatively charged glutamate residues, which increase their affinity and reinforce their presentation by dendritic cells to CD4+ T cells in the context of HLA-DQ2 or DQ8 molecules. Dendritic cells present deaminated gliadin peptides to CD4+ T cells. Activated gluten-reactive CD4+ T cells produce high levels of proinflammatory cytokines, including interferon (IFN)-γ, interleukin (IL)-21, and IL-15, thereby inducing T-helper (Th) cell type 1. This promotes inflammatory effects, including lamina propria mononuclear cell secretion of matrix metalloproteinases responsible for degradation of the extracellular matrix and basement membrane and increased cytotoxicity of CD8+ intraepithelial lymphocytes, as well as favoring apoptosis of lamina propria enterocytes. Furthermore, activated CD4+ T cells, through the production of Th2 cytokines, drive the activation and clonal expansion of B cells, resulting in the production of anti-tTG antibodies.

When gluten peptides cross the lamina propria, they undergo deamination by tTG and become rich in negatively charged glutamate residues. This process increases the affinity and reinforces the presentation of gluten peptides by dendritic cells (a type of antigen-presenting cell) to CD4+ T cells in the context of HLA-DQ2 or HLA-DQ8 molecules [13,33]. Antigen-presenting cells present deaminated gliadin peptides to CD4+ T cells, which are activated to produce proinflammatory cytokines, chiefly interferon (IFN)-γ, interleukin (IL)-17A, and IL-21, thereby inducing T-helper (Th) cell type 1 [1]. IFN-γ promotes inflammatory effects by activating matrix metalloproteinase secretion from lamina propria mononuclear cells, which are responsible for the degradation of the extracellular matrix and basement membrane, as well as increasing the cytotoxicity of CD8+ intraepithelial lymphocytes [13]. The intraepithelial lymphocytes move to the intraepithelial compartment and destroy the villous structure of the small intestine by inducing cytotoxicity and apoptosis, with the participation of IL-15 [13,30]. The same T cells also induce a Th2 response-releasing cytokine that activates the clonal expansion of B cells, with the subsequent production of anti-tTG autoantibodies, the serological hallmark of CD [13,30,31,32,33] (Figure 3).

Serological tests may be performed in the clinical setting, and a positive result supports the diagnosis; however, no single test is 100% specific for CD, and diagnostic accuracy varies considerably between laboratories [5]. These tests consist of measuring tTG-IgA while on a regular gluten-containing diet and concurrent measurement of total IgA if the patient had not previously been tested for IgA deficiency. Patients with elevated tTG-IgA levels should undergo upper gastrointestinal endoscopy with duodenal biopsy [5]. Intestinal biopsy has been a central test to confirm the diagnosis of CD since the late 1950s [34], as it demonstrates the histological changes associated with the disease, such as increased intraepithelial lymphocytes, villous atrophy, and crypt hyperplasia, classified according to the Marsh [35] or simplified Corazza classification [36]. In children, in an exceptional situation, a no-biopsy strategy using solely a positive serological test could be accepted to confirm the diagnosis, but it is applicable when the family agrees, with high levels of tTG-IgA (>10× the upper limit of normal should be obligatory) and a positive endomysial antibody in a second blood sample [5,37].

A GFD is the only effective therapy for patients with CD. A systematic review supported the role of strict adherence to a GFD in controlling symptoms, improving quality of life, and decreasing the risk of complications [5,6]. Patients and their families should understand the role of a GFD in achieving high dietary adherence [38,39]. Ideally, information on GFDs should be provided in collaboration with dietitians [34]. However, patients with CD report a reduced quality of life because of dietary restrictions [38], and some data suggest that 25% of patients with CD are not satisfied with the information offered by their physician, and some report dissatisfaction with the information provided by the dietitian [40].

Once the diagnosis is made, follow-up should be initiated to reinforce dietary adherence, provide psychological support, and carry out clinical and laboratory evaluation, including associated autoimmune conditions (mainly type 1 diabetes, thyroid disorders, and liver diseases) [17]. Dietary adherence can be assessed by a self-reported GFD, measuring celiac-specific antibodies such as anti-tTG, and performing endoscopic and histological reassessment; however, the real need and ideal time to perform invasive tests are controversial [17]. In adults, neither symptoms nor serology are reliable for predicting damage to the small intestine. Serum anti-tTG is correlated with adherence to the diet; however, it has a low sensitivity for persistent villous atrophy [41]. Some other serum biomarkers, which are not yet used in clinical practice, could be considered as monitoring tools in addition to traditional markers to assess adherence to a GFD and as non-invasive tests to correlate with the severity of mucosal damage. These include anti-Saccharomyces cerevisiae (ASCA) IgG and/or IgA, which are present in a high proportion in patients with CD (59%) with a decrease in ASCA IgA after DIG [42], and IgA anti-actin antibodies, which are also present in a high percentage of patients with untreated CD (higher in Marsh 3b-c than 1-3a) and disappear after 1 year of DIG (and Marsh 0 assessed by second intestinal biopsy), showing a high correlation with histological damage and recovery [41]. Practically, the cause of CD has not been established, and there are no specific tests for their proper diagnosis and assessment. No drugs are currently available to treat this condition. However, some studies have investigated the involvement of miRNAs in disease pathogenesis.

## 3. MicroRNA

**Figure 4:**
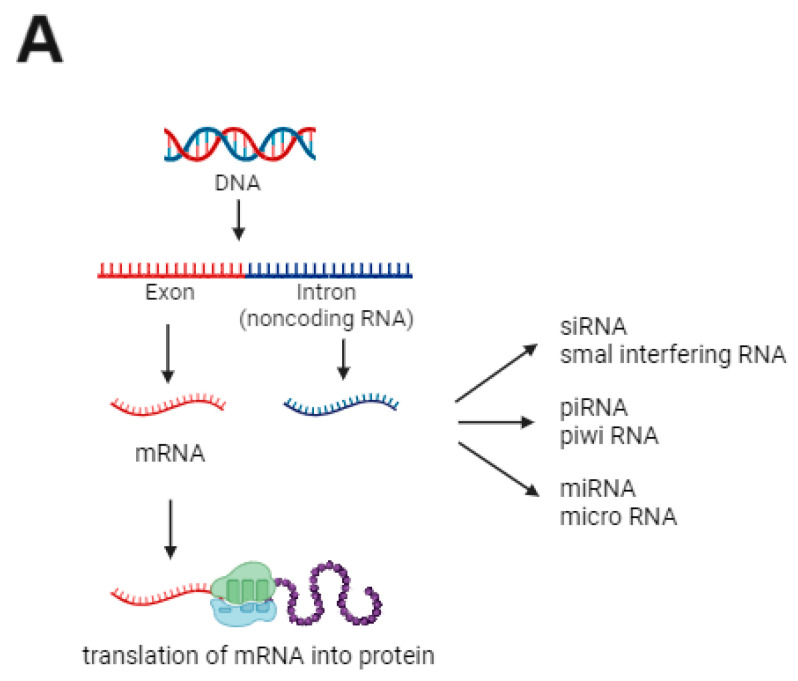

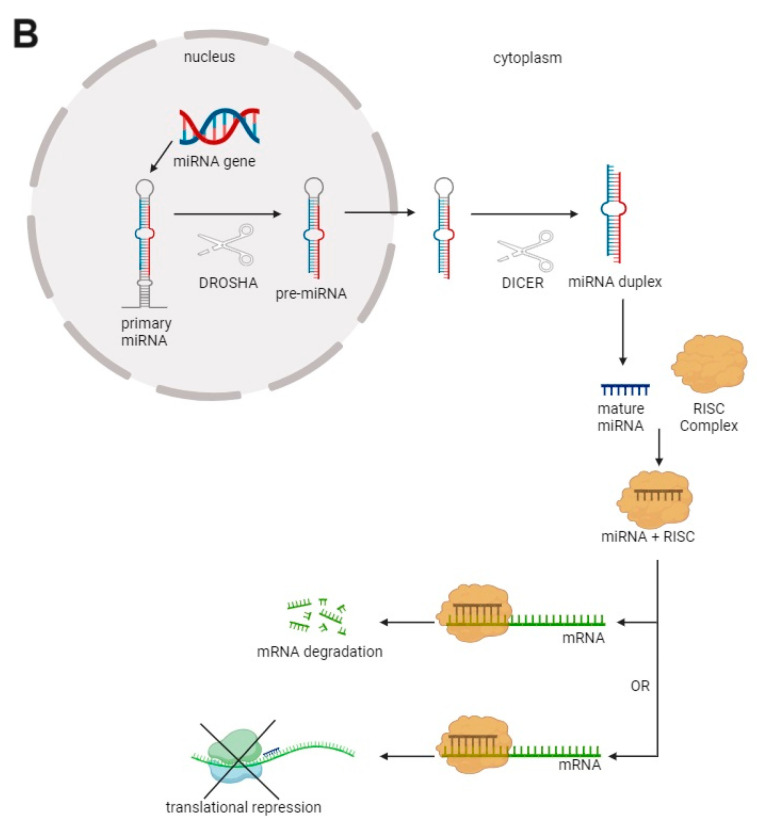
MicroRNA (miRNA) biogenesis and mode of action. (**A**) DNA contains regions called exons (nucleic acid coding sequences), which originate from messenger RNAs (mRNA) and are later transcribed into proteins, and introns (noncoding sequences), which are important to the origin of the miRNA; (**B**) inside the cell nucleus, miRNA is transcribed to primary miRNA and then to precursor miRNA (pre-miRNA) by the microprocessor complex Drosha/DGCR8 (DROSHA). The pre-miRNA is then transferred to the cell cytoplasm. Dicer participates in the second processing step to produce miRNA duplexes. The duplex is separated, and one strand is usually selected as the mature miRNA, whereas the other strand is degraded. The mature miRNA is loaded into an RNA-induced silencing complex (RISC) and regulates gene expression by binding to mRNA and causing its degradation or blocking its translation into proteins.

miRNAs are a class of small, single-stranded, noncoding RNA molecules of approximately 18–25 nucleotides that inhibit the translation of multiple mRNAs, thereby silencing their target genes [43]. It is estimated that there are >700 miRNA genes in the human genome, and hundreds of targets may be controlled [2,44]. miRNAs are believed to control approximately 30% of gene expression [43,45] by binding to mRNAs and inducing their degradation or repression, thereby upregulating or downregulating the expression of specific genes [43,46] (Figure 4).

miRNAs play key roles in the differentiation and function of the intestinal epithelium by regulating gene expression in both normal and pathological states, including inflammatory and autoimmune diseases [47]. Currently, since there are not enough available data on the molecular factors underlying CD, it is necessary to assess the profile and functions of miRNAs in these patients [16].

Dysregulation of the immune system may involve alterations in protein expression, either at the transcriptional or post-transcriptional level. In such cases, miRNAs may play a fundamental role. Studies carried out in patients with CD with different clinical features have shown an alteration of miRNA expression analyzed from tissue (miR-31-5p, miR-192-3p, miR-192-5p, miR-194-5p, miR-197, miR-338-3p, miR-551a, miR-551-5p, miR-551b-5p, miR-638, and miR-1290) and blood/plasma (miR-21, miR-146a, miR-155, miR-326, and miR-125b) samples [7,8,9,43,45]. Some of these studies compared patients with CD to healthy individuals [7,8,9,43,45] and individuals with different clinical symptoms [45], the severity of intestinal damage [45], GFD, or gluten ingestion [7,8,9]. Some studies have assessed miRNA profiles in blood samples [7,8,9], whereas others have assessed miRNA profiles in duodenal biopsy samples [7,43,45].

These studies have shown that the miRNA profile is involved in a cascade of immune mechanisms and consequences of inflammatory disorders in CD, such as cell proliferation and differentiation, regulatory T-cell development, innate immune response, activation of the inflammatory cascade, focal adhesion, T-cell commitment, tissue transglutaminase synthesis, and the cell cycle [2,16].

The greatest concern is that there are still few data with different methodologies that cannot produce strong conclusions. Various factors can alter miRNA profiles because different sample sites, such as plasma, urine, feces, and tissues, do not have the same miRNA profile [2].

## 4. Evaluation of miRNA in Tissue Samples from Individuals with CD

The literature on the relationship between CD and tissue or circulating miRNA profiles is scarce. The analysis of miRNAs in humans with CD is relatively new and was first described in 2014 [45]. Since then, few studies have been performed with duodenal mucosa evaluation in different groups with varied symptoms and mucosal damage, such as those on GFDs or gluten consumption. Eleven miRNAs were significantly associated (*p* < 0.05) with CD: miR-31-5p, miR-192-3p, miR-192-5p, miR-194-5p, miR-197, miR-338-3p, miR-551a, miR-551-5p, miR-551b-5p, miR-638, and miR-1290 [7,43,45] (Table 1).

Magni et al. [43] performed a study including miRNA analysis from biopsies of patients with active CD with moderate histological lesions (Marsh 3A-B) and severe injury (Marsh 3C), patients with CD on a GFD and in remission (Marsh 0–1), and a control group of healthy individuals. After miRNA profile evaluation, comparing miRNA expression in controls versus Marsh 3C CD, they found downregulated expression of seven miRNAs in patients with active CD: miR-638, miR-192-5p, miR-483-3p, miR-31-5p, miR-517c, miR-338-3p, and miR-197 (*p* < 0.01). This indicates that the pattern of miRNA expression in the duodenal mucosa of adult patients with CD differs from that of healthy control individuals [43]. Subsequently, the study evaluated each group of patients (active CD Marsh 3A-B, active CD Marsh 3C, and control) and observed a significant reduction in four miRNAs in the biopsy samples of patients with CD: miR-192-5p, miR-338-3p and miR-197 (Marsh 3A-B and 3C versus control), and miR-31-5p (Marsh 3C versus control) [43]. miR-192-5p expression, which is related to cell proliferation and apoptosis with high expression in the normal epithelium of the intestine, was significantly reduced in patients with CD Marsh 3A-B, with a profound decrease in patients with CD Marsh 3C (approximately a 32% reduction), compared with controls, showing a correlation with the severity of the intestinal lesion [43]. These four significantly reduced miRNAs in active CD were tested during in vitro stimulation with gliadin exposure in healthy controls and patients with remission CD on a GFD before and after stimulation [43]. miR-192-5p was the only reported miRNA with a significant reduction compared to that in GFD biopsies before and after in vitro stimulation with gliadin exposure [43]. The levels of miR-192-5p increased in the controls after stimulation; however, in patients with CD on a GFD, a 42% reduction was observed. This finding suggested that innate immunity plays a key role in the altered response to gluten observed in the intestinal mucosa of patients with CD because the expression of miR-192-5p had a different response in patients with and without CD, even if both patient groups had normal mucosa [43].

Vaira et al. [45] included 64 individuals distributed in five groups: untreated “classical” CD, untreated “atypical” CD with iron deficiency anemia, treated and asymptomatic GFD with normal mucosa, treated and asymptomatic GFD with atrophic mucosa, and healthy individuals (control group) [45]. The comparison between healthy controls and patients with CD (“classical” or “anemia” type) revealed a significant level (*p* < 0.05) of overexpression of miR-1290 and miR-551b-5p and underexpression of miR-31-5p and miR-192-3p in both types of CD presentations [45]. When comparing different presentation types (“anemia” versus “classical”), miR-194-5p and miR-551-5p were less expressed, and miR-638 expression was upregulated but only in the anemia-type group (*p* < 0.01) [45]. miR-192-3p and miR-551a were analyzed in four groups: patients on a GFD who had CD with normalization of histology, patients who had CD without treatment, patients on a GFD with persistent duodenal mucosal damage, and healthy individuals (control group). Both miRNAs showed decreased expression in untreated CD and duodenal atrophy in patients on a GFD compared to a control group [45]. miR-192-3p showed a small increase (no statistical significance) in patients with duodenal normalization, and miR-551a expression was increased (*p* < 0.05) when comparing CD on a GFD with persistent duodenal mucosal damage with that in patients on a GFD with mucosal normalization [45]. These results show that the miRNA-192/194 cluster is significantly deregulated in patients with classical- or anemia-type CD, but its expression recovers in patients on a GFD with normalization of the duodenal mucosa [45]. miR-55a showed a similar behavior and was re-expressed in individuals with normalization of the mucosa. Both miRNAs have been linked to extracellular matrix remodeling [45]. miR-192 is modulated by transforming growth factor-β, an important agent in CD and matrix remodeling [45]. Thus, miRNAs could be important new biomarkers to distinguish patients with CD with diverse clinical manifestations because miRNAs are differentially expressed in patients with different clinical manifestations.

## 5. Evaluation of Circulating miRNAs in Patients with CD

The mechanism of acquisition of circulating miRNA samples is the least invasive, as they can be collected from different body fluids (blood, plasma, and urine). Moreover, these molecules are highly stable in circulation and under diverse laboratory conditions. Therefore, many studies investigating the profile of circulating miRNAs in CD have been published [7,8,9,10,11]. However, few studies have been conducted on adults [7,8,9].

Only five circulating miRNAs were altered: miR-21, miR-146a, miR-155, miR-326, and miR-125b [7,8,9]. These miRNAs were analyzed in the context of different sources, including blood, plasma, monocytes, and peripheral blood mononuclear cells (PBMCs), and the groups were divided into the following: patients with CD at diagnosis, those on a GFD during disease remission, and healthy individual controls (Table 2).

Bascuñán et al. [7] analyzed tissues and circulating miRNAs in three groups: active CD during diagnosis, patients with CD on a GFD and in histologic remission (Marsh 0), and a healthy control group. In addition, they performed in vitro gliadin stimulation in two groups: CD at the time of diagnosis and patients with CD on a GFD. Four miRNAs were analyzed based on previous studies: miR-146a, miR-155, miR-21, and miR-125b. CD-active and CD-inactive versus controls showed increased expression of miR-146a, miR-155, and miR-21 in PBMCs, and of miR-155 in monocytes (all *p* < 0.001), suggesting that a GFD does not affect miRNA expression [7]. After gliadin stimulation of PBMCs and monocytes, miR-146a and miR-155 expression was upregulated in active and inactive patients [7]. These data suggest that changes in the expression of some miRNAs are related to disease rather than treatment. Therefore, some of these miRNAs can be used as biomarkers to diagnose CD in patients on a GFD before confirming the disease. There were no changes in miRNA expression in the intestinal mucosa, suggesting that intestinal and systemic regulation and dysregulation may follow different pathways [7].

Circulating miRNA expression was studied by Domsa et al. [8], who analyzed four miRNAs based on previous reports (miR-192-5p, miR-194-5p, miR-449a, and miR-638) in three different groups: active CD during diagnosis, patients with inactive CD on a GFD, and a control group with healthy individuals. The CD at diagnosis and GFD groups had an increased level of miR-194-5p (very close to the significance values of *p* = 0.0510 and *p* = 0.0671, respectively) compared to the control group [8].

Nafari et al. [9] assessed the relationship between IL-17, a proinflammatory interleukin, and miR-326, the expression of which is associated with the pathogenesis of autoimmune disorders such as multiple sclerosis, type 1 diabetes, and autoimmune thyroiditis [9]. Blood samples were collected from patients with inactive CD on a GFD for >1 year and from healthy individuals. They expected that the expression of miR-326 and IL-17 would decrease in individuals with GFD; however, miR-326 expression was significantly downregulated in CD-inactive patients on a GFD versus healthy controls (*p* = 0.001), contradicting their hypothesis, whereas IL-17 was highly expressed in patients with CD despite adherence to a GFD (*p* = 0.002) [9].

## 6. Final Considerations and Future Perspectives

CD is an immune-mediated enteropathy triggered by the ingestion of “gluten” in genetically predisposed patients. The “classical” description of CD was made >100 years ago; since then, many improvements have been made, particularly related to genetic predisposition, pathogenesis, forms of clinical presentation (“classical”, “atypical”, and even asymptomatic), and diagnostic criteria (serological markers and histology alterations in small intestinal samples). Nevertheless, the etiology of the disease has not yet been established, and there are no specific tests for proper diagnosis. To date, the only available treatment is adherence to a strict GFD.

The upregulation or downregulation of miRNA expression can affect several pathways related to CD pathogenesis. This review shows that miRNA expression can suppress or stimulate pathways related to CD pathogenesis by regulating basic cellular functions, such as cell proliferation and differentiation, regulatory T-cell development, innate immune response, activation of the inflammatory cascade, focal adhesion, T-cell commitment, tissue transglutaminase synthesis, and cell cycle. Furthermore, the interaction between miRNAs and oxidative stress might play a role in the pathophysiology of CD, as recently demonstrated by Pelizzaro et al. [48].

In conclusion, CD is the most prevalent enteropathy; however, many carriers of this condition do not have a proper diagnosis. Moreover, diagnosis and follow-up are invasive, and the only available treatment is long-term adherence to a GFD. Thus, identifying miRNAs and their related effects on CD could open new possibilities for diagnosis, prognosis, and follow-up biomarkers, and miRNAs could be used to develop specific therapies aimed at inhibiting CD pathophysiological pathways through miRNA suppression or stimulation.

The scientific literature on the expression of circulating and/or intestinal miRNAs in CD is still scarce, with a few small studies using a myriad of miRNA analyses and a wide spectrum of methodologies, thereby limiting conclusive remarks. In addition, miRNA analysis is expensive. Both these aspects suggest that further studies, ideally with a large cohort of patients with CD, should be performed to determine the potential use of miRNAs in CD treatment.

## Figures and Tables

**Table 1:** miRNAs in tissue samples (duodenal biopsy) of the adult population.

Included Studies	Population (Number)	Material	Increased (↑) miRNAs	Decreased (↓) miRNAs
Magni et al., 2014 [43]	31 individuals for miRNA expression: (2 groups): - 21: Active CD (9 Marsh 3A-B + 12 Marsh 3C) - 10: control group AND 14 individuals for in vitro gliadin exposure for 4 and 24 h and fibroblast culture analysis (2 groups): - 9: Remission CD on GFD - 5: control group Both before and after stimulation for 4 and 24 h	Intestinal mucosa	No significant changes	Control vs. active CD: ↓ miR-192-5p ↓ miR-31-5p ↓ miR-338-3p ↓ miR-197 AND Remission CD on GFD fibroblast before vs. after gliadin exposure: ↓ miR-192-5p
Vaira et al., 2014 [45]	64 individuals (5 groups): - 12: “classical” CD - 11: “anemia” CD - 18: GFD normal mucosa - 11: GFD atrophic mucosa - 12: healthy controls 6 patients from the CD group + 4 from the control group were tested “in vitro” before and after gliadin exposure	Intestinal mucosa and fibroblast culture	Control vs. “classical” CD: ↑ miR-551b-5p ↑ miR-1290 Control vs. “anemia” CD: ↑ miR-551b-5p ↑ miR-1290 “Classical” vs. “anemia” CD: ↑ miR-638 GFD atrophic mucosa vs. GFD normal mucosa ↑ miR-551a	Control vs. “classical” CD: ↓ miR-31-5p ↓ miR-192-3p Control vs. “anemia” CD: ↓ miR-31-5p ↓ miR-192-3p “Classical” vs. “anemia” CD: ↓ miR-194-5p ↓ miR-551b-5p Control vs. active CD and Control vs. GFD atrophic mucosa: ↓ miR-192-3p ↓ miR-551a

miRNA, microRNA; CD, celiac disease; GFD, gluten-free diet.

**Table 2:** miRNAs in the blood samples of the adult population.

Included Studies	Population (Number)	Material	Increased (↑) miRNAs	Decreased (↓) miRNAs
Bascuñán et al., 2020 [7]	30 individuals (3 groups): - 10: CD at diagnosis - 10: GFD -10: control group	Plasma, PBMCs, monocytes (and intestinal mucosa)	CD at diagnosis vs. control and GFD vs. control: in monocytes↑ miR-146a; ↑ miR-155; ↑ miR-21. in PBMCs ↑ miR-155. in plasma ↑ miR-155; ↑ miR-21; ↑ miR-125bCD at diagnosis and GFD before and after in vitro gliadin stimulation. in PBMCs and monocytes ↑ miR-146a; ↑ miR-155. only in PBMCs ↑ miR-21.	No significant changes
Domsa et al., 2022 [8]	58 individuals (3 groups): - 15: CD at diagnosis - 33: GFD - 10: control group	Blood	CD at diagnosis vs. control: ↑ miR-194-5p (very close to the significant value *p* = 0.0510) GFD vs. control: ↑ miR-194-5p (close to the significant value *p* = 0.0671)	No significant changes
Nafari et al., 2022 [9]	80 individuals (2 groups): - 40: GFD - 40: control group	Blood	No significant changes	GFD vs. controls ↓ miR-326

miRNA, microRNA; CD, celiac disease; GFD, gluten-free diet; PBMC, peripheral blood mononuclear cells.
